# *Caenorhabditis elegans* as a model system for studying non-cell-autonomous mechanisms in protein-misfolding diseases

**DOI:** 10.1242/dmm.013011

**Published:** 2014-01

**Authors:** Carmen I. Nussbaum-Krammer, Richard I. Morimoto

**Affiliations:** Department of Molecular Biosciences, Rice Institute for Biomedical Research, Northwestern University, 2205 Tech Drive, Evanston, IL 60208, USA.

**Keywords:** *Caenorhabditis elegans*, Cell non-autonomous proteotoxicity, Prion-like spreading

## Abstract

*Caenorhabditis elegans* has a number of distinct advantages that are useful for understanding the basis for cellular and organismal dysfunction underlying age-associated diseases of protein misfolding. Although protein aggregation, a key feature of human neurodegenerative diseases, has been typically explored *in vivo* at the single-cell level using cells in culture, there is now increasing evidence that proteotoxicity has a non-cell-autonomous component and is communicated between cells and tissues in a multicellular organism. These discoveries have opened up new avenues for the use of *C. elegans* as an ideal animal model system to study non-cell-autonomous proteotoxicity, prion-like propagation of aggregation-prone proteins, and the organismal regulation of stress responses and proteostasis. This Review focuses on recent evidence that *C. elegans* has mechanisms to transmit certain classes of toxic proteins between tissues and a complex stress response that integrates and coordinates signals from single cells and tissues across the organism. These findings emphasize the potential of *C. elegans* to provide insights into non-cell-autonomous proteotoxic mechanisms underlying age-related protein-misfolding diseases.

## *C. elegans* as a model system to study the toxicity of disease-associated proteins

The nematode *Caenorhabditis elegans* has many unique characteristics that render it an attractive model system, and it has been instrumental in the discovery of fundamental biological processes in development, neurobiology and aging. Importantly, the animal is transparent, thus allowing for *in vivo* tracking of cells over time and visualization of fluorescently tagged proteins in the living organism (Brenner, 1974; Chalfie et al., 1994). The entire cell lineage of all 959 adult somatic cells of the *C. elegans* hermaphrodite (the predominant sex) has been traced and the morphology and synaptic connections of all 302 neurons have been mapped (Sulston, 1983; Sulston et al., 1983; White et al., 1986). The worm has a relatively short life cycle (~3.5 days), fast reproduction cycle with a high progeny number (~300), short lifespan (~2 weeks) and can be easily cultivated on agar plates or in liquid media, which makes it amenable to a wide variety of high-throughput manipulations. In addition, the *C. elegans* research community benefits from its powerful genetic and imaging toolbox, which continues to be expanded and improved (Xu and Kim, 2011; Boulin and Hobert, 2012). It was the first multicellular organism to have its genome sequenced, and ~80% of the proteins encoded in the *C. elegans* genome are conserved from worms to vertebrates (Lai et al., 2000). Furthermore, basic cell biological principles and neuronal signaling pathways are conserved between *C. elegans* and humans.

Like other invertebrate animal models, the cell biology of *C. elegans* mirrors that of humans, yet the organism is amenable to a wide variety of well-established genetic, molecular and biochemical analyses. Because of its short life cycle, the animal is particularly useful for addressing questions about aging. Moreover, a number of protein-misfolding disorders (PMDs), which comprise several age-related neurodegenerative diseases, have been successfully modeled in the animal, usually by transgenic expression of the respective human disease genes or associated pathological fragments. The first *C. elegans* PMD model to be generated was based on transgenic expression of the Aβ peptide, a component of amyloid plaques associated with Alzheimer’s disease (AD) (Link, 1995), and many other transgenic models followed (Faber et al., 1999; Satyal et al., 2000; Parker et al., 2001; Morley et al., 2002; Kraemer et al., 2003; Lakso et al., 2003; Kuwahara et al., 2006; Park and Li, 2008; Gidalevitz et al., 2009; Wang et al., 2009; Ash et al., 2010; Dosanjh et al., 2010; Teixeira-Castro et al., 2011). The transgenes used are typically fluorescently tagged and expressed under promoters that allow tissue-specific expression in the body wall muscle cells, intestine or neurons. Most disease-linked proteins are aggregation-prone and tend to self-assemble into aggregate species that can be easily visualized in the living animal. When the protein is expressed in muscle or neuronal cells, toxicity of these proteins typically results in tissue damage and subsequent paralysis or uncoordinated movement, respectively, because all *C. elegans* somatic cells are post-mitotic and have no self-renewal capacities. The readily detectable phenotypes of aggregation and toxicity can then be used for subsequent genome-wide screens for genetic enhancers or repressors to discover previously unknown disease genes, or to decipher pathways of known disease genes (Nollen et al., 2004; van Ham et al., 2008; Silva et al., 2011; Treusch et al., 2011; Lejeune et al., 2012; Lim et al., 2012). Several studies using such transgenic animals have led to the important discovery that genes that prolong lifespan also restore proteostasis (protein homeostasis), providing a link between aging and proteotoxicity and thus a plausible reason for the age-dependent onset of neurodegenerative diseases (Morley et al., 2002; Hsu et al., 2003; Morley and Morimoto, 2004; Cohen et al., 2006). Furthermore, *C. elegans* has been successfully used for the discovery and evaluation of drugs (Calamini et al., 2011; Fatouros et al., 2012; Lublin and Link, 2013).

Of note, in contrast to recent observations in humans and mouse models (as outlined in the next section), all the transgenic *C. elegans* models seem to exhibit only tissue-specific toxicity, such that disease proteins that are expressed in body wall muscle cells cause muscle cell dysfunction and related phenotypes, and, similarly, neuronal expression of the transgenes causes a failure of neuronal function. This implies that the transgenes involved act in a cell-autonomous manner, i.e. only the cells that express the gene exhibit the specific phenotype. By contrast, a gene is said to have a non-cell-autonomous effect if other cells show the same phenotype without expressing the corresponding gene. Further evidence of cell-autonomous toxicity in *C. elegans* has been obtained by co-expressing tissue-specific temperature-sensitive (ts) mutant proteins with polyglutamine (polyQ; associated with trinucleotide-repeat disorders) and superoxide dismutase 1 [SOD1; associated with amyotrophic lateral sclerosis (ALS)] (Gidalevitz et al., 2006; Gidalevitz et al., 2009). Ts mutants can be used as proteostasis indicators, because they are metastable and highly dependent on the cellular protein-folding machinery. A disruption of the cellular folding homeostasis (e.g. by expression of polyQ) exposes the ts mutant phenotype at restrictive temperatures. Intriguingly, the ts mutant phenotypes were only detectable when polyQ or SOD1 were expressed in the same tissue, but not upon expression in a different tissue (Gidalevitz et al., 2006; Gidalevitz et al., 2009).

These experiments in *C. elegans* would suggest that disease-associated proteins act cell-autonomously, i.e. do not exert transacting effects on other tissues. However, non-cell-autonomous effects cannot be ruled out on the basis of early *C. elegans* studies. In part this is because of the systemic and heritable effects of RNA interference (RNAi), which was the most commonly used approach until recent years. Tools for tissue-specific knockdowns have only recently been developed (Calixto et al., 2010). Another reason is that both neuronal and muscle failure leads to motility defects, and this phenotype has been used as the read-out for many of the genetic screens.

Given that there is growing evidence that protein aggregates actually do exhibit non-cell-autonomous toxicity, a better knowledge about the underlying mechanisms is necessary for the prospect of more effective treatments. The following sections will highlight research on the complex proteotoxicity phenotypes underlying numerous neurological disorders and discuss the recent use of *C. elegans* as a genetic model system to study non-cell-autonomous mechanisms in PMDs and stress responses.

## Non-cell-autonomous effects in PMDs

In many neurodegenerative diseases, most of which are triggered by protein misfolding, including AD, Parkinson’s disease (PD), ALS and Huntington’s disease (HD), only a subset of neurons are vulnerable to proteotoxic stress and damage, despite the ubiquitous expression of the disease-associated protein(s) (Jackson, 2013). Thus, specific neuronal populations are affected in different neurodegenerative disorders. For example, dopaminergic neurons in the substantia nigra are affected in PD (Forno, 1996), motor neurons in ALS (Rowland and Shneider, 2001), medium spiny neurons in the striatum in HD (Ferrante et al., 1985) and Purkinje neurons in the cerebellum in spinocerebellar ataxias (SCAs) (Garden and La Spada, 2008). However, there is accumulating evidence that direct damage of these vulnerable neurons by misfolded protein species might not be the main reason for their selective degeneration. This is highlighted by studies showing that the exclusive expression of a mutant protein in specific neuronal cell types affected in disease does not always lead to the expected disease phenotypes (Clement et al., 2003; Gu et al., 2005; Gu et al., 2007; Yamanaka et al., 2008a). For example, mutant SOD1 (associated with ALS) expression only in motor neurons does not lead to any detectable pathology in mice (Lino et al., 2002). Furthermore, cell therapy often does not show the desired positive effect because grafted neuronal cells – despite being young and healthy – eventually die when transplanted into brains affected by neurodegeneration (Li et al., 2008; Desplats et al., 2009; Cicchetti et al., 2011).

Linked to this is the finding that cell types that are not killed upon expression of the aberrant protein can still influence and aggravate toxicity in neighboring cells. The various contributions of neighboring cells to disease onset and progression have been demonstrated by studies in which the disease protein is selectively silenced or expressed in non-neuronal cells (Raeber et al., 1997; Garden et al., 2002; Clement et al., 2003; Shin et al., 2005; Yazawa et al., 2005; Beers et al., 2006; Boillée et al., 2006; Custer et al., 2006; Di Giorgio et al., 2007; Yamanaka et al., 2008b). For example, the overexpression of α-synuclein (associated with PD) in oligodendrocytes can cause degeneration of neurons and glia cells in a mouse model of multiple system atrophy (Yazawa et al., 2005), and the presence of wild-type (WT) SOD1 in non-motor neurons substantially delays onset of motor neuron degeneration in ALS mice (Yamanaka et al., 2008a). A plethora of mechanisms have been suggested to underlie this non-cell-autonomous toxicity in PMDs, including diminished trophic and nutrient support, glutamate excitotoxicity, and activation of microglia, which mediate inflammatory responses (reviewed in Lobsiger and Cleveland, 2007; Ilieva et al., 2009; Sambataro and Pennuto, 2012). These studies demonstrate that almost all PMDs are caused by the damage of diverse cell types that collectively contribute to the loss of selective neurons by non-cell-autonomous mechanisms. Vice versa, one may conclude that therapeutic interventions that target non-neuronal or even peripheral tissues might have a critical impact on disease progression (Ilieva et al., 2009; Sambataro and Pennuto, 2012). However, more accessible model systems are necessary to study the molecular basis and identify potential therapeutic targets within these complex interrelations.

## Using *C. elegans* to study non-cell-autonomous effects in PMDs

Expression of polyQ-containing protein in *C. elegans* neurons has revealed neuron-dependent variation in protein solubility upon pan-neuronal expression of polyQ at the pathological threshold of 40 glutamine residues (Brignull et al., 2006). Whereas polyQ40 remained soluble in ALM mechanosensory neurons, the BDU interneurons, the HSN motor neurons and the CAN neurons, fluorescence recovery after photobleaching (FRAP) analysis revealed the presence of both soluble and immobile protein species in the motor neurons of the ventral (VNC) and dorsal (DNC) nerve cord (Brignull et al., 2006). This suggests that the latter motor neurons might be more sensitive to polyQ40. However, it remains to be determined whether this was due to cell autonomous or non-cell-autonomous effects, for instance by expressing polyQ proteins of different lengths in distinct subsets of neurons.

Subsequent studies have provided evidence that an imbalance in signaling in the motor neurons can influence proteostasis in postsynaptic body wall muscle cells. Defective γ-aminobutyric acid (GABA) signaling or increased acetylcholine (ACh) signaling causes a general imbalance in protein homeostasis in postsynaptic muscle cells and led to the premature appearance of polyQ35 aggregates, which usually appear in older worms (Garcia et al., 2007). Interestingly, a manipulation of the balance between ACh and GABA, under the threshold of excitotoxicity, had the opposite effect. The balance between ACh and GABA signaling seems to be critical, with an extreme overstimulation leading to proteotoxic stress and a physiological enhancement of ACh signaling being proteoprotective. A genome-wide RNAi screen identified GEI-11, a negative regulator of cholinergic receptor activity at the neuromuscular junction (NMJ), as an enhancer of polyQ aggregation in body wall muscle cells (Silva et al., 2011). Downregulation of *gei-11* activated the heat shock response (HSR) and heat shock transcription factor 1 (HSF-1)-dependent induction of cytosolic chaperone expression, restoring proteostasis (Silva et al., 2013). These studies revealed the importance of fine-tuning neuronal signaling within a critical physiological threshold, which might represent a new target to restore proteostasis across tissues.

## Prion-like mechanisms in PMDs

A well-known but only recently revisited feature of several neurodegenerative diseases is a characteristic spread of disease pathology during disease progression (Braak et al., 1993; Braak et al., 2002; Brundin et al., 2010). Although a specific subset of neurons are highly vulnerable in each disease and constitute the site of disease onset (see above), other cells are affected as the disorder advances during aging. Multiple lines of evidence have suggested that the toxic protein species might transit across cells and tissues, thus invading adjacent cells to propagate their aggregation-prone conformation in a prion-like process (Polymenidou and Cleveland, 2012). Prions are self-propagating aggregates that account for the infectious nature of transmissible spongiform encephalopathies (TSEs) in mammals and the epigenetic inheritance of certain traits in yeast (Wickner, 1994; Prusiner, 1998).

It is uncertain whether all PMD-related proteins will turn out to have characteristics of bona fide prions, because there might exist several levels of prion-like behaviors. For example, prions autocatalytically self-assemble into amyloid structures, and numerous studies have revealed similarities in assembly pathways of both non-prion amyloidogenic proteins and prions (Soto, 2003). Aggregation of many PMD-associated proteins follows a crystallization-like process, called nucleated or seeded polymerization (Jarrett and Lansbury, 1993; Scherzinger et al., 1999; Wood et al., 1999). However, this feature alone does not fulfill all of the criteria for prion-ness. For example, prions replicate by spreading to naïve cells and seeding the conversion of the soluble isoforms. Consistent with this concept, amyloid protein A (AA; associated with AA amyloidosis), Aβ, α-synuclein, SOD1, tau (associated with taupathies and AD), huntingtin (associated with HD) and TDP-43 [associated with frontotemporal lobar degeneration with ubiqitin-positive inclusions (FTLD-U) and ALS] all have the capacity to seed aggregation of soluble homotypic proteins *in vitro* and *in vivo*. The addition of *in vitro* fibrillated proteins to cultured cells has been shown to seed soluble endogenous proteins in a sequence-specific manner (Yang et al., 2002; Danzer et al., 2009; Frost et al., 2009; Ren et al., 2009; Münch et al., 2011; Wang et al., 2012; Nonaka et al., 2013). Induced aggregates have been shown to propagate (with varying efficiency) during cell division and from cell to cell in co-culture experiments (Clavaguera et al., 2009; Frost et al., 2009; Krammer et al., 2009a; Ren et al., 2009; Hansen et al., 2011; Münch et al., 2011; Nonaka et al., 2013). Moreover, inclusions of aggregated α-synuclein were found in neurons grafted into the brain of PD patients, suggesting a potential transmission of the disease from host to grafted tissue (Li et al., 2008; Desplats et al., 2009). Furthermore, direct injection of protein aggregates can induce or at least accelerate aggregation of homologous proteins in animal models of amyloid diseases (Lundmark et al., 2002; Meyer-Luehmann et al., 2006; Clavaguera et al., 2009; Eisele et al., 2010; Luk et al., 2012; Morales et al., 2012).

Ultimately, prions spread from host to host and can infect new individuals. Thus far, evidence of transmission between hosts has only been observed with AA, where inoculation of the protein from feces of cheetahs accelerated AA amyloidosis in mice (Zhang et al., 2008). To date, there is no epidemiological evidence that PMDs other than prion diseases are infectious among humans. However, given the high incidence of some of these diseases, a spreading mechanism under certain circumstances cannot be excluded.

A better understanding of the cellular pathways that underlie cell-to-cell transmission is necessary for the development of novel therapeutics. However, currently available models to investigate prion biology include unicellular organisms, such as yeast, tissue culture cells or mammalian animal models, which are of limited suitability to investigate these mechanisms in detail. Yeast prions naturally disseminate from mother to daughter cells within the cytosol during cell division, which does not involve transport across membrane borders. Cell culture models lack the natural environment of an intact organism and, in mouse models, cells and proteins are difficult to track without intervention. The amenability of *C. elegans* to genetic manipulation and its transparency provide the potential to discover the mechanisms underlying prion-like propagation because it allows cell-to-cell transmission of aggregation-prone proteins in a living metazoan organism to be monitored in real time.

## Modeling prion-like spreading in *C. elegans*

**Fig. 1.**
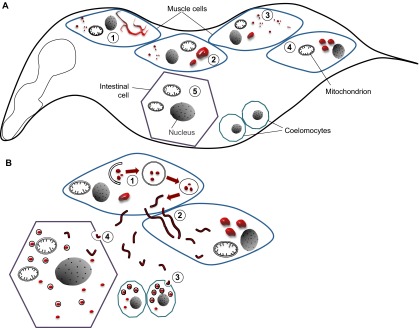
**Cell-autonomous and non-cell-autonomous characteristics of the *C. elegans* prion model.** (A) Expression of the well-characterized glutamine/asparagine (Q/N)-rich prion domain NM of the cytosolic yeast prion protein Sup35 (red) in body wall muscle cells leads to the formation of diverse foci with different properties. Large fluorescent foci of accumulated NM were analyzed by fluorescence recovery after photobleaching (FRAP) and categorized into fibril-like immobile aggregates (containing non- diffusing protein; (1) and spherical mobile aggregates (containing slowly diffusing protein; (2). Small foci were not assessed by FRAP and consist of both small aggregates and vesicles (3). Fragmented mitochondria were observed in muscle cells (4) and in non-expressing tissues (5) by TEM, indicating cell-autonomous and non-cell autonomous toxicity. (B) NM colocalizes to autophagy-related vesicles that transport the prion domain from the site of expression to adjacent tissues. NM is targeted by the autophagy-lysosomal systems (1), which allows the misfolded protein to enter a topologically different compartment, from the cytosol to membrane-bound vesicles. Instead of being degraded, the prion domain accumulates in LMP-1::GFP-positive tubular structures that are transported within and between cells (2). The prion domain was also detected in vesicles of coelomocytes (immune cells) (3) and the intestine (4), indicating that it is released from muscle cells and endocytosed by these tissues.

Because there are no known prion proteins in *C. elegans*, we recently used the well-characterized glutamine/asparagine (Q/N)-rich prion domain NM of the cytosolic yeast prion protein Sup35 to develop a *C. elegans* prion model (Nussbaum-Krammer et al., 2013) (Fig. 1). This domain is necessary and sufficient for prion propagation in yeast (Ter-Avanesyan et al., 1994). Strikingly, this prion model exhibited a toxicity phenotype that was substantially different than the *C. elegans* models that transgenically express disease-associated proteins for PMDs (Link, 1995; Morley et al., 2002; Park and Li, 2008; van Ham et al., 2008; Gidalevitz et al., 2009). Aggregation of NM led to cell-autonomous and non-autonomous toxicity, i.e. as well as affecting the body wall muscle cells expressing the transgene, the cellular morphology of neighboring tissues was also disrupted. NM was targeted for autophagy; however, instead of being degraded, it accumulated in lysosomes (marked by LMP-1, the worm homolog of lysosome-associated membrane protein 1). Intriguingly, NM-containing vesicles did not just conglomerate within a single cell, but could be observed, in real time, being transported within and between cells. These findings demonstrated that cytosolic aggregation-prone proteins can exhibit prion-like spreading (corroborating recent findings, described below) and, intriguingly, this is driven by vesicle transport.

Until recently, cell-to-cell transmission of infectious protein entities and induction of self-propagating protein aggregates in the recipient cells had only been fully established for the mammalian prion protein, which is a glycosylphosphatidylinositol (GPI)-anchored membrane protein. Although NM aggregates were shown to be able to propagate in murine neuroblastoma cells during cell division, similar to in yeast (Krammer et al., 2009b), Speare et al. had demonstrated that GPI anchoring facilitated spreading of NM from cell to cell (Speare et al., 2010). In contrast, the recent demonstration that cytosolic NM aggregates can also invade neighboring cells in primary cell culture and organotypic brain slices, and induce heritable self-perpetuating aggregates in the recipient cells, clearly shows that the mammalian cytosolic environment promotes prion propagation (Hofmann et al., 2013). Thus, the cytosolic yeast prion domain can propagate in mammalian cells, adapt to different requirements for propagation and fulfill the infectious cycle of a bona fide prion (Krammer et al., 2009b; Hofmann et al., 2013).

Our recent data indicated that altering the topological environment of a cytosolic protein by taking it up into membrane-bound vesicles via autophagy facilitates cell-to-cell transfer (Nussbaum-Krammer et al., 2013). Given that numerous disease-related cytosolic proteins have been described as substrates of the autophagy-lysosomal pathway, these results imply that this mechanism might be the basis of amyloid infectivity in general. Indeed, recent work using amyloid precursor protein (APP) transgenic mice that are deficient for autophagy demonstrated that the release of Aβ and extracellular plaque formation is mediated by autophagy (Nilsson et al., 2013). Moreover, α-synuclein was shown to be secreted in exosomes, and the secretion was increased when lysosomal degradation was blocked (Danzer et al., 2012).

Lysosomes have recently emerged as multifunctional organelles for which protein degradation is just one of its roles. They have been shown to be exocytosed for cell membrane reparation (Reddy et al., 2001) and to be transferred from endothelial progenitors to stressed endothelial cells via tunneling nanotubes to reconstitute the lysosomal pool and restore cell viability (Yasuda et al., 2011). Because lysosomes in aging post-mitotic tissue usually build up as lipofuscin (Terman and Brunk, 1998), it is unlikely that lysosomes are just released into the extracellular space, if they cannot degrade their content. Rather, it is tempting to speculate that the expression of the prion domain might trigger and hijack a cellular ‘rescue-me’ response that would lead to the transfer of lysosomes containing the infectious proteins to an unaffected cell. In line with this, it has been repeatedly observed that specific cells can recognize stressed cells and subsequently initiate the transfer of cytosolic content, such as lysosomes or mitochondria, to rescue the damaged cell (Spees et al., 2006; Yasuda et al., 2011; Pasquier et al., 2013). Although this transfer seems to be selective from non-stressed to stressed cells, prions might be transported in the other direction to infect naïve cells (Gousset et al., 2009). Further studies of *C. elegans* should help discover the mechanisms that regulate the transfer of these vesicles containing prion-like proteins.

## Non-cell-autonomous HSR regulation and cell-to-cell communication of proteostasis in *C. elegans*

**Fig. 2.**
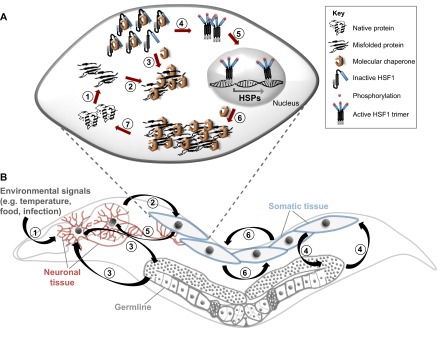
**Cell-autonomous and non-cell-autonomous regulation of the HSR.** (A) Cell-autonomous regulation of the HSR in isolated eukaryotic cells. The regulation of the HSR is directly coupled to the folding requirements in each cell. An acute exposure to heat shock or other proteotoxic stresses leads to the rapid accumulation of misfolded proteins (1). The recruitment of molecular chaperones by misfolded proteins (2) leads to the de-repression of heat shock transcription factor HSF-1 (3). Binding of Hsc70 and Hsp90 negatively regulates HSF-1 by keeping it in its monomeric cytosolic form. Upon increased accumulation of misfolded proteins, these chaperones preferentially bind to their non-native and misfolded clients and release HSF-1, which then gets phosphorylated and trimerizes (4), translocates into the nucleus (5), and activates the transcription of inducible chaperones and other components of the proteostasis network (6). Elevated levels of chaperones help to clear and refold protein aggregates and restore proteostasis (7). (B) Non-cell-autonomous regulation of the HSR in *C. elegans*. In metazoans, the HSR of individual cells and tissues is regulated at multiple levels. Neurons sense and integrate diverse environmental signals (1) and control the HSR in somatic tissues (2). The germ line communicates with neurons (and vice versa) (3) and non-neuronal tissues (4) to favor reproduction and suppress stress responses. Neurons also receive signals from other somatic tissues that can result in behavioral changes (5). Crosstalk between different somatic tissues then coordinates the HSR at the organismal level (6).

The increasing number of disease-related misfolded proteins that exhibit non-cell-autonomous effects has led to speculations that metazoan organisms might have a stress response that functions beyond the immediately affected cell. The regulation of the HSR has been historically studied as a cell-autonomous process in yeast and metazoan tissue culture cells, in which HSF-1 is activated by titration of inhibitory chaperones through the accumulation of misfolded proteins (Ananthan et al., 1986; Morimoto, 1998; Åkerfelt et al., 2010) (Fig. 2A). However, during aging and upon chronic expression of aggregation-prone proteins, as in neurodegenerative diseases, the HSR is not efficiently activated, suggesting the presence of additional layers of regulatory control.

At the whole-animal level, the HSR is regulated by a non-cell-autonomous mechanism (Fig. 2B). In *C. elegans*, the thermosensory neurons (AFD neurons), which sense and integrate ambient temperature to regulate thermotaxis behavior, were shown to regulate the HSR in non-neuronal somatic tissues (Prahlad et al., 2008) and to control their cellular response to misfolded proteins (Prahlad and Morimoto, 2011). Although the absence of AFD function (due to a mutation in the gene encoding the receptor-type guanylyl cyclase GCY-8 that is exclusively expressed in AFD neurons) led to a significantly diminished HSR, the same mutant animals coped much better with chronic proteotoxic stress caused by polyQ aggregates. Thus, under acute conditions, the AFD neurons are necessary to mount a robust HSR, whereas, under chronic proteotoxic stress, the same neurons negatively control the HSF-1-dependent expression of molecular chaperones. A similar phenomenon was identified with the G protein-coupled receptor (GPCR) thermal receptor 1 (GTR-1), which is expressed in *C. elegans* chemosensory neurons (Maman et al., 2013). Indications that a similar non-cell-autonomous control of the HSR might take place in other organisms have arisen from studies in rats, where neuroendocrine signaling mediated by the hypothalamic-pituitary-adrenal axis leads to the activation of HSF-1 and HSP70 in adrenal tissue (Blake et al., 1991; Fawcett et al., 1994).

Other signals, such as the availability of food, are also integrated into the HSR regulatory network in *C. elegans* (Prahlad et al., 2008). Hence, the manipulation of the HSR by neurons is not only dependent on temperature or accumulation of misfolded proteins, but also on other regulatory circuits that are interconnected with the sensing of the HSR. Although this complex arrangement seems counterproductive in the face of neurodegenerative diseases (because this seems to impede a constitutive activation of a presumably protective HSR), it raises the hope that there might be a way to manipulate this system, once we have a better understanding of the underlying mechanisms of all pathways involved.

Similar to the HSR, the unfolded protein response (UPR) of the endoplasmic reticulum (UPR^ER^) and the mitochondrial UPR (UPR^mito^) were also previously thought to be regulated at the cellular level. Studies in mammalian cells revealed that the accumulation of unfolded proteins in the ER directly activates the ER stress sensor, the transmembrane protein kinase and endonuclease IRE-1 (Gardner and Walter, 2011); likewise, in *C. elegans*, the UPR^mito^ transcription factor ATFS-1 is activated when mitochondrial import is blocked (Nargund et al., 2012). It has been shown in *C. elegans* that when neurons encounter ER or mitochondrial stress, the same stress response is turned on in non-neuronal cells, even though these cells did not directly suffer from an increase in misfolded proteins. For example, knocking down cytochrome *c* oxidase-1 subunit Vb/COX4 (*cco-1*; a component of the electron transport chain in mitochondria) specifically in neurons leads to mitochondrial stress in both neurons and the intestine (Durieux et al., 2011). Similarly, neuronal expression of a constitutively active form of the ER-stress-response-associated transcription factor XBP-1 (XBP-1s) was able to rescue the age-dependent decline of stress resistance and increase longevity, by activating the UPR^ER^ in distal cell types through a mechanism that involves the release of neurotransmitters by small clear vesicles (SCVs) (Taylor and Dillin, 2013). Furthermore, several recent studies revealed evidence of non-cell-autonomous crosstalk between the innate immune response and UPR^ER^. OCTR-1, a catecholamine GPCR for the biogenic amine neurotransmitter octopamine, is exclusively expressed in ASH and ASI sensory neurons and negatively regulates the innate immune response in non-neuronal tissues by suppressing the expression of *abu* genes, a family of genes shown to be involved in the ER stress response when the canonical UPR is blocked (Urano et al., 2002; Sun et al., 2011). OCTR-1 also regulates the canonical UPR at the organismal level by blocking XBP-1-mediated resistance to pathogens (Sun et al., 2012). This regulatory process is only effective during adulthood and not during development, which further indicates that the organism constantly integrates multiple signals into a final systemic response.

These findings highlight the importance of the nervous system as a central regulator of diverse animal stress responses. Neurons, however, are not the only mediators of such non-cell-autonomous regulatory networks. There is accumulating evidence from studies in *C. elegans* of feedback circuits that signal from peripheral tissues to trigger behavioral responses. Non-neuronal cells are able to sense ambient temperature in an HSF-1-dependent manner and influence neuronal thermotactic behavior through an estrogen signaling pathway (Sugi et al., 2011). In addition, somatic tissue that experiences a life-threatening disruption of key cellular processes such as translation, respiration and protein turnover stimulates a food-avoidance behavior that is usually induced by pathogens and toxins and requires serotonergic and JNK kinase signaling pathways (Melo and Ruvkun, 2012). Thus, *C. elegans* possesses a surveillance network that monitors important cellular machineries, which triggers an aversion response upon detection of cellular perturbations (Melo and Ruvkun, 2012).

Additional support for tissue-wide communication was shown for DAF-21, the *C. elegans* HSP90 homolog that functions as an organismal proteostasis regulator (van Oosten-Hawle et al., 2013). Expression of a metastable temperature-sensitive mutant myosin heavy chain B (UNC-54), a muscle-specific client protein of DAF-21, resulted in upregulation of *DAF-21* mRNA levels not only in muscle cells but also in cells that do not express UNC-54, such as the intestine. Overexpression of DAF-21 in the intestine or neurons led to non-cell-autonomous rescue of UNC-54 misfolding in the muscle, analogous to a muscle-specific overexpression of DAF-21. Consistent with these results, an imbalance of DAF-21 levels between different tissues could also modulate the HSR in a global manner. This non-cell-autonomous transcriptional regulation of HSP90 levels between somatic tissues was independent of neuronal control, but dependent on the FOXA transcription factor PHA-4 (van Oosten-Hawle et al., 2013). Thus, HSP90 seems to be a regulatory hub in the surveillance of organismal proteostasis. It remains to be determined whether HSP90 is the only member of the proteostasis network whose expression is coordinated between multiple tissues to balance the HSR across the entire animal.

Although the FOXO transcription factor DAF-16 was not shown to be a mediator of transcellular chaperone signaling, it can also act non-cell-autonomously to direct stress-gene expression. DAF-16 activity in the endoderm ameliorates the motility defects caused by expression of Aβ peptide in body wall muscle cells, possibly by MDT-15-dependent intercellular lipid signals (Zhang et al., 2013).

There is also evidence that the HSR can be restricted to specific tissues. A genome-wide RNAi screen for regulators of the HSR in *C. elegans* identified genes that are required for the efficient induction of the HSR function in all tissues, in addition to negative regulators of the HSR that, when knocked down, induce the HSR in a tissue-selective manner (Guisbert et al., 2013). For example, the systemic knock down of proteasomal subunits induces the expression of an *hsp70*-promoter::GFP transcriptional reporter only in the intestine and spermatheca, whereas knockdown of TriC (also known as CCT) chaperonin subunits induced the reporter only in muscle cells. Intriguingly, both of these complexes are ubiquitously expressed and are likely to be important for protein folding and degradation in all tissues. However, the specialized function of each tissue might result in a higher dependence on certain members of the proteostasis network (Guisbert et al., 2013). Furthermore, each tissue presumably has a very distinct proteostasis network, optimized for their specific proteome. Hence, the proteostasis network is also regulated in a tissue-specific way, according to the unique functional requirement in each tissue. Further work will be necessary to determine which mechanisms influence the decision between a cell-autonomous and non-cell-autonomous regulation of the proteostasis network across different tissues in an animal.

In addition to neurons, the germ line has been shown to be important for the non-cell-autonomous regulation of somatic physiology, evidenced by the recent identification of signals that control organismal proteostasis originating from germline stem cells (Shemesh et al., 2013). Several different downstream effector pathways are influenced by germline stem cell signaling to modulate the somatic stress responses in favor of reproduction.

Taken together, these studies suggest that multicellular animals have evolved an elaborate system that integrates multiple external (from the environment) and internal (from soma and germ line) stimuli in order to coordinate a systemic response or favor a tissue-selective response to stress. Importantly, the *C. elegans* model also has its limitations, and its strengths are also its weaknesses. Owing to its simplicity, human disease pathology is not completely recapitulated – 302 neurons can only go so far – and some genes and pathways are not conserved in the nematode. Therefore, it is questionable whether all findings in the worm can be translated to humans. Similar studies have yet to be conducted in other, more complex model organisms to test a broader relevance of these findings for human stress biology.

## Conclusions and outlook

Although the HSR can be triggered cell autonomously, there is growing evidence that stress responses and proteostasis of individual cells are not regulated independently within a multicellular organism, but rather integrate and coordinate information from and with their environment. This communication occurs at several levels: between neighboring cells within the same tissue, between different tissues within the same organism, as well as between an organism and its external environment, mediated by sensory organs (Fig. 2). Likewise, proteotoxicity is not exclusively cell autonomous, suggesting that the organism integrates signals from several cells and tissues to coordinate a concerted stress response to adequately cope with more complex diseases. Although such a conjoined response might be beneficial under most conditions to ensure survival of single cells, it could also be detrimental under other circumstances. For example, during chronic PMDs, a prolonged stress response (by suppressing the negative regulation by neurons) would be rather advantageous.

Taken together, the recent observations of non-cell-autonomous proteotoxicity and cell-to-cell spreading of misfolded proteins in neurodegenerative diseases, and our lack of understanding of the underlying mechanisms, demonstrate the importance for more accessible biological systems, exemplified by *C. elegans*, to study this complex interaction between cells and tissues in an organism upon proteostatic challenge. How do protein aggregates in one tissue influence proteostasis in neighboring cells? Do all aggregation-prone proteins get transmitted between cells? What are the protein and cellular requirements for transmission? What are the components of the organism-wide proteostasis network? Which factors are only acting to implement a tissue-specific response, and how do all of the factors communicate? *C. elegans* now offers all the tools necessary to address these questions, and future studies in this exceptional animal model will contribute significantly to the study of age-related protein-misfolding diseases.
